# Exploring the Impact of Artificial Intelligence and Machine Learning in the Diagnosis and Management of Esthesioneuroblastomas: A Comprehensive Review

**DOI:** 10.7759/cureus.62683

**Published:** 2024-06-19

**Authors:** Raj Patel, Tadas Masys, Refat Baridi

**Affiliations:** 1 Otolaryngology - Head and Neck Surgery, Loyola University Chicago Stritch School of Medicine, Chicago, USA; 2 Medicine, Loyola University Chicago Stritch School of Medicine, Chicago, USA; 3 Oncology, Silver Cross Hospital, Oak Brook, USA

**Keywords:** machine learning models, artificial intelligence in radiology, machine learning healthcare data, artificial intelligence(ai), esthesioneuroblastoma and seizures

## Abstract

Esthesioneuroblastomas (ENBs) present unique diagnostic and therapeutic challenges due to their rare and complex clinical presentation. In recent years, artificial intelligence (AI) and machine learning (ML) have emerged as promising tools in various medical specialties, revolutionizing diagnostic accuracy, treatment planning, and patient outcomes. However, their application in ENBs remains relatively unexplored. This comprehensive literature review aims to evaluate the current state of AI and ML technologies in ENB diagnosis, radiological and histopathological imaging, and treatment planning. By synthesizing existing evidence and identifying gaps in knowledge, this review aims to showcase the potential benefits, limitations, and future directions of integrating AI and ML into the multidisciplinary management of ENBs.

## Introduction and background

**Figure 1:**
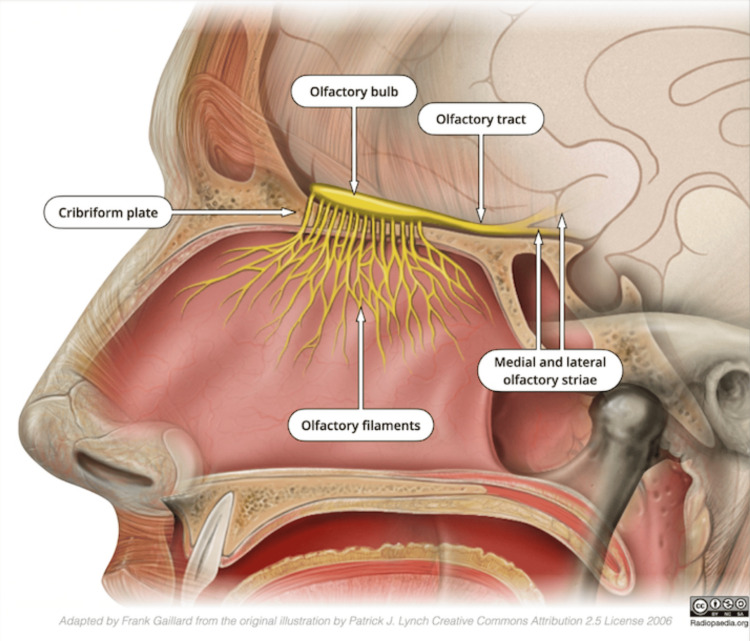
Anatomy of the sinonasal cavity and proximal anatomical structures. Image Credit: Adapted by Frank Gaillard from the original illustration by Patrick J. Lynch, Creative Commons Attribution 2.5 License 2006 [3]

An esthesioneuroblastoma (ENB), also referred to as an olfactory neuroblastoma, is a rare malignancy of the sinonasal tract [1]. This tumor originates from specialized sensory neuroepithelium olfactory cells, which are located in the upper nasal cavity, in proximity to the superior portion of the septum, superior nasal concha, the roof of the nose, and the cribriform plate of the ethmoid sinus (Figure 1) [1,2,3]. ENBs arise from primitive neuroectodermal cells, which are neural precursor cells (NPCs). These precursor cells grow and give rise to immature neuroblasts. ENBs develop when these neuroblasts divide and grow in an uncontrolled manner [3]. ENBs account for 2-6% of all intranasal malignancies [4,5]. To put the rarity of ENB cases into perspective, there have been less than 700 cases documented in the United States and fewer than 400 unique cases reported globally. ENB is equally prevalent between male and female patients between the ages of 40 and 60 [4]. 

Of note, there is a significant lack of depth of information on certain patient groups, populations, and risk factors associated with ENB [6,7]. A few risk factors have been linked to ENBs; however, they are based on observation and are not yet conclusive. Like many cancers, ENB development is associated with aging due to cellular changes that occur throughout an individual's life. Cells experience senescence, where they have reduced proliferative ability; aging can increase the chance of oxidative DNA damage and reduce the ability to respond to DNA damage. These cellular processes all play a role in the development of ENBs in the aging population [5,6]. Additionally, some sources suggest that exposure to wood dust and nickel compounds is carcinogenic and can lead to ENB development [6,7]. There seems to be no significant understanding of the hereditary patterns of ENBs or whether there is an interplay between genetic and environmental components [7]. 

Currently, there is active research being undertaken including the discovery of possible chromosomal changes or mutations that may play a role in the etiology of ENBs. Some studies have confirmed chromosomal gains in 7q11 and 20q, and deletions in 2q, 5q, 6p, 6q, and 18q [6]. Further investigation can provide significant utility for karyotyping patients suspected to have ENBs and can direct the treatment and management for them. Alongside chromosomal discoveries, molecular genetic investigations have been made where two transcription factors have been studied that are associated with ENB. The transcription factors, SATB2 and GATA3, have been shown to be correlated to sinonasal neuroendocrine neoplasms [8]. Molecular investigation of the genetic components of ENBs can be utilized to identify diagnostic markers when a patient is suspected of having a potential ENB.

The five-year survival rate for ENB patients after treatment ranges from 50-80%, depending largely on factors such as the stage of the cancer at the time of diagnosis, the clinical treatment strategy, and the overall health of the patient [9]. ENBs have the capability of stimulating angiogenesis, the formation of new blood vessels, to ensure an adequate blood supply for their growth and survival [1,9]. Angiogenesis allows ENBs to acquire invasive properties, enabling them to grow locally around adjacent structures such as the sinuses, cribriform plate, and ethmoid bones [1,9]. 

The likelihood of tumor recurrence after treatment can range between 20-50% [9,10]. The possible reasons for the recurrence of ENB include incomplete surgical resection, advanced malignant stages leading to metastasis, and even failure of adjuvant therapies. ENBs can exhibit metastatic properties, with the rate of metastasis ranging from 10-30% [10,11]. Common metastatic locations include regional lymph nodes, followed by distant sites like the lungs, liver, bones, and brain [10,12]. Once an ENB becomes metastatic, the prognosis and treatment options change drastically because the tumor is no longer localized [4,6]. Thus, more systemic considerations are required to treat it. 

**Figure 2:**
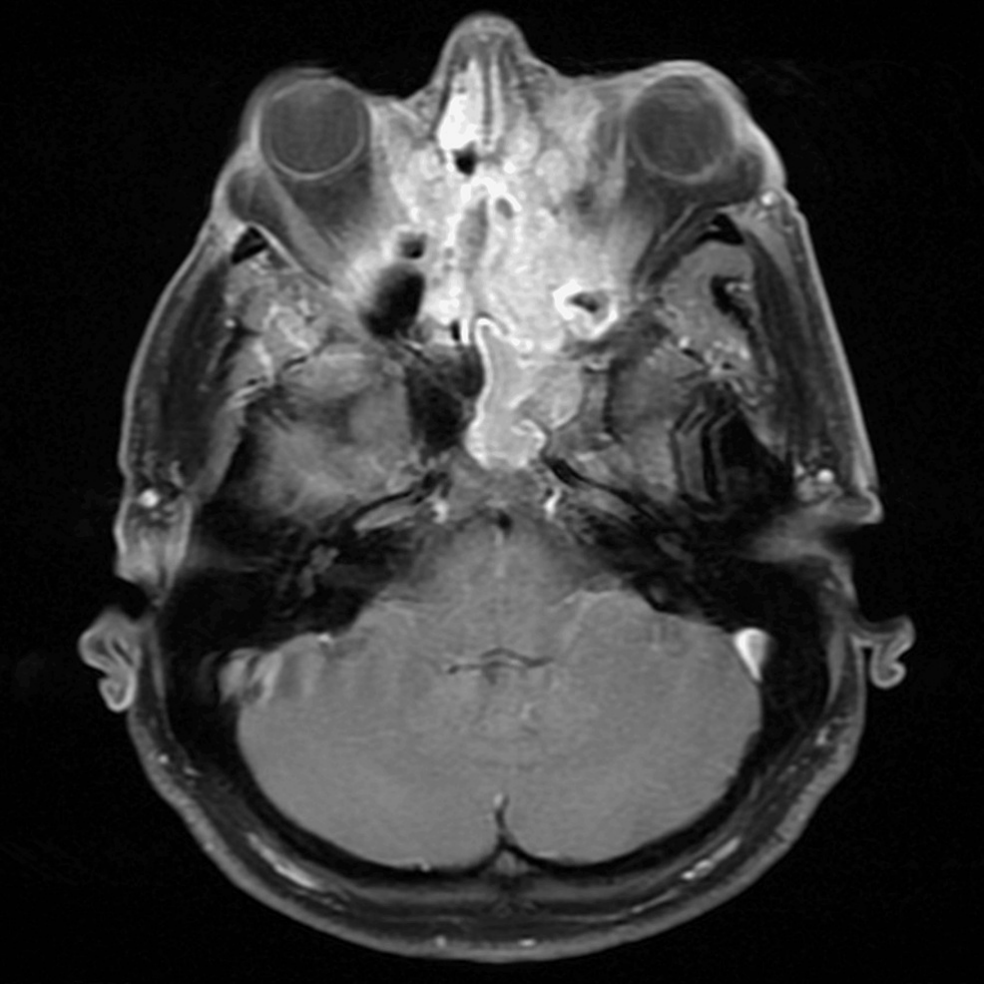
CT image of an esthesioneuroblastoma, depicted as a large enhancing mass centered on the midline of the anterior cranial fossa and involving both orbits. Image Credit: Frank Gaillard, 2010 [14]; Creative Commons License

The symptoms that are seen in patients with ENBs are non-specific due to the location of the tumor near critical structures of the head and neck [1,4,13]. Symptoms can vary depending on the tumor’s size, location, and the extent of its spread. Some symptoms include nosebleeds, breathing problems, congestion, headache, eye pain, loss of smell, and neurological problems [1,4]. CT scans and MRI tend to be the most prevalent imaging techniques to visualize ENBs in the sinonasal cavity. ENBs pose a challenge when analyzing a CT or MRI due to a lack of unique phenotypic appearance (Figure 2) [14]. These non-specific symptoms render diagnosing ENBs challenging, as they can easily be mistaken for more common diseases that affect the nasal cavity [6].

Proper treatment and management of ENBs require a multidisciplinary approach [15]. The options of surgery, radiation therapy, chemotherapy, and active surveillance, may all be considered when creating a treatment plan for a patient [1,15]. Surgical resection of the tumor is the primary treatment for an ENB, especially for localized tumors. The goal of surgery is to achieve a negative tumor margin while preserving the nearby anatomical structures of the head and neck [16]. Depending on the characteristics of the tumor, various surgical techniques may be employed, including endoscopic resection, open surgery, or a combination of both [16]. Radiation therapy consists of utilizing an external beam of high-energy wavelength to localize damage to the tumor [17]. This ensures minimal proximal damage to the anatomy. This therapy may be used before or after surgery but is typically administered postoperatively to eliminate any remaining tumor cells that could not be resected [16,17]. Chemotherapy may be combined with surgical approaches and/or radiation therapy, particularly in more advanced metastatic ENBs. Chemotherapy is typically administered before surgery to shrink the tumor and facilitate its removal, or after surgery to destroy any remaining cancer cells [18]. Additionally, active surveillance can be a useful strategy in managing ENBs. Depending on the severity of symptoms and the growth rate of the tumor, monitoring these clinical presentations can assist in making a proper treatment plan for the patient [1,4]. The clinical staging of ENB treatment varies from patient to patient. The decision to administer therapy before or after surgical treatment is determined by factors such as clinical staging, tumor size, location, and potential metastasis [1,4,18]. Unfortunately, the literature provides limited information on the optimal clinical staging for treating patients with ENB. 

Due to the low prevalence of ENBs, their complex anatomical locations, and non-specific symptoms early in the tumor’s progression, diagnosing ENBs is challenging. Especially in the early stages, an ENB is more likely to be misdiagnosed or mistaken for other benign nasal or sinus conditions. Advanced-stage tumors, however, are more readily recognized due to their aggressive nature and associated symptoms [1,6]. Thus, to treat and manage an ENB effectively before the patient experiences worsening symptoms, more accurate diagnostic tools must be implemented. To address this need, Artificial Intelligence (AI) and Machine Learning (ML) are emerging as promising solutions that could revolutionize the early detection and diagnosis of challenging conditions such as an ENB.

AI and ML are transformative technologies and just the surface has been scratched regarding their abilities to reshape numerous industries, including healthcare. AI refers to the ability of machines to perform tasks that typically require human intelligence. This encompasses learning, reasoning, problem-solving, perception, and language understanding. ML, a subset of AI, involves algorithms and statistical models that are designed to learn from data and make decisions or predictions, without being explicitly programmed for each specific task [19]. The process involves collecting and preparing data, choosing and training a model on this data, and then testing the model's performance. If the model performs well, it can be deployed to address real-world problems. In the medical field, for instance, it may assist with diagnostics, treatment planning, and personalized patient care [19,20]. To ensure it remains effective, the model is regularly updated with additional data sets and continuously monitored.

**Figure 3:**
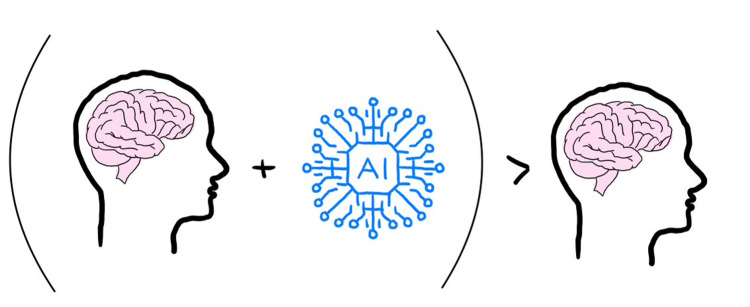
Friedman's Fundamental Theorem of Biomedical Informatics depicting the synergistic utilization of AI technologies with healthcare clinicians. Image Credit: Author Raj Patel

In the context of integrating AI technologies within the medical field, the term “augmented intelligence” is particularly appropriate. This term emphasizes the enhancement and augmentation of human clinician capabilities, rather than their “artificial” replacement [21]. This encapsulates a symbiotic relationship in which AI supports and enhances the clinical insights of physicians, rather than substituting their expertise. Therefore, it is important to highlight that AI and ML are designed to augment, not replace, the critical role of physicians in healthcare (Figure 3). In a conceptual adaptation of Friedman’s Fundamental Theorem of Biomedical Informatics, it is posited that clinicians who effectively use information technology in healthcare will outperform not only those who avoid its use but also the outcomes of AI and ML models operating independently [22]. AI and ML can process vast amounts of data far beyond human capability in medicine, identifying patterns and providing insights that can significantly improve diagnostic accuracy and treatment efficacy [19,23]. However, the irreplaceable value of human judgment, empathy, and ethical considerations in medical practice guarantees that clinicians will remain integral to healthcare delivery [24]. Ultimately, this highlights the collaboration between AI technologies and clinicians, fostering a more precise and comprehensive approach to the diagnosis and management of conditions such as an ENB.

In healthcare, AI and ML are redefining the way medical professionals diagnose, treat, and manage diseases. These technologies analyze vast amounts of data quickly and with precision, enhancing clinicians' abilities to make more informed decisions [19]. There are several AI models that are currently being used in the diagnosis of cancer cells in medicine. In healthcare, AI and ML applications include medical imaging analysis, where AI algorithms excel at analyzing images from X-rays, CT scans, MRI scans, and mammograms [23,25]. These algorithms and statistical models can aid in detecting abnormalities that may indicate the presence of cancer, supporting radiologists in making accurate diagnoses. Additionally, pathology image analysis will use algorithms and statistical models to aid in examining tissue samples from biopsies [23]. These algorithms can detect and differentiate between malignant and non-malignant cells, helping pathologists classify tumors more effectively [23,26]. Furthermore, through ​​genomic analysis, AI is instrumental in analyzing genomic data to identify mutations linked to cancer [27]. This ML analysis can forecast cancer risks, influence treatment decisions, and pinpoint new therapeutic targets [23,27]. Another AI model that is currently being used includes clinical decision support systems. These AI-powered systems integrate diverse patient data, medical images, pathology results, genomic data, and clinical records to assist in diagnostic and treatment decisions. They tailor recommendations to individual patients based on evidence-based guidelines [23]. Additionally, AI is used in radiomics to extract high-dimensional quantitative features from medical images. This information can predict tumor behavior, treatment response, and patient outcomes, providing insights beyond what the naked eye can see [27,28,29].

The integration of AI and ML in ENB cases offers a plethora of potential benefits. ML could potentially enhance diagnostic accuracy and streamline the treatment planning process by learning from patterns in vast datasets. AI's ability to rapidly analyze complex datasets not only speeds up the interpretation of medical images and pathology slides but may also accelerate early cancer detection and timely treatment initiation, which may significantly improve patient outcomes [28]. Furthermore, AI-driven genomic analysis allows for more personalized treatment approaches, tailoring the treatment plan specifically to a patient’s unique genetic profile, thus potentially improving the efficacy of therapies [20,23]. These technologies are not just tools to aid clinicians but potential game-changers in the fight against cancer, making a previously unimaginable level of personalized and precise medicine possible.

## Review

Methodology

A manual literature search was conducted to find key, relevant papers discussing ENBs, AI, and ML. The literature search was performed on Pubmed using the following keywords: esthesioneuroblastoma, artificial intelligence in healthcare, and machine learning in healthcare.

Inclusion criteria for key papers on ENBs encompassed case reports, case series, and comprehensive studies. Only studies that discussed cases or the pathophysiology of ENBs were included to ensure proper characterization and analysis of the tumor. Papers published within the last five years on ENBs were considered to ensure the inclusion of recent information.

The inclusion criteria for key papers on AI and ML in clinical settings encompassed comprehensive reviews. Papers that did not address AI and ML in the healthcare diagnostic or management process were excluded. This approach allowed for the inclusion of specific AI and ML modalities used in healthcare, providing detailed information on their utilization in medicine. Due to the recent emergence of AI and ML technologies in a clinical setting, there was no restriction on the publication date of the journals included. 

AI and ML in ENB diagnosis

Radiological Imaging

The integration of AI and ML into radiological imaging has marked a significant advancement in the field of oncology, particularly in the diagnostic accuracy and characterization of tumors using CT, MRI, and positron emission tomography-CT (PET-CT) scans [28]. These technological advancements, through their systematic algorithms and deep learning capabilities, are not only enhancing the precision of radiological imaging but are also revolutionizing the way tumors are detected, monitored, and managed.

The application of AI and ML in medical imaging, particularly CT and MRI, has dramatically improved the detection and characterization of tumors. AI algorithms are trained to recognize subtle patterns and anomalies that may not be evident to the human eye, enabling earlier and more accurate detection of malignancies [23,28]. In MRI and CT scans, for example, AI-driven tools analyze the presence, shape, size, symmetry, density, texture, and even genetic makeup of tumors when integrated with other sources like genomic data [23,27,30]. This detailed characterization helps determine the nature of the tumor, whether it is benign or malignant, its stage, and potential aggressiveness, which are all essential for choosing the right treatment strategy [23]. This capability is crucial for the early diagnosis and staging of cancer, which in turn guides treatment planning and improves prognosis. MRI, known for its superior soft tissue contrast compared to CT, benefits significantly from AI in terms of image segmentation and tumor boundary identification [23,30]. Due to ML excelling at processing and analyzing large volumes of imaging data quickly, ML algorithms can effectively differentiate between various types of tissues and identify tumor margins, which are critical for surgical planning and radiation therapy [23]. With this in mind, ML algorithms may provide an advantage when it comes to diagnosing ENBs early on. Consequently, the deployment of AI in medical imaging not only enhances diagnostic accuracy but also significantly streamlines radiological workflows.

A recent study on paranasal sinus disease detection using automated machine learning (AutoML) demonstrates AutoML's capacity to optimize diagnostic processes, achieving a precision rate of 92.8% and an accuracy of 92% in identifying disease presence through MRI scans. This example underlines the potential of AI to reduce the workload on radiologists by accurately automating the detection and initial diagnosis stages, which could be particularly transformative in the early detection and management of ENBs [31]. As mentioned before, a challenge that ENBs pose is the difficulty of diagnosing and identifying characteristics early in their development. By providing multiple scans of early-state ENBs, ML can generate algorithms to pick up on minor patterns to provide certainty of an ENB diagnosis. This can mitigate clinical error where surgeons mistake ENBs for other sinonasal malignancies and proper care can be provided to patients. Furthermore, AI-enhanced medical imaging analysis can track changes in tumor size and response to treatment over time, providing valuable data for assessing patient progress. PET-CT scans, which combine metabolic and anatomical imaging, offer a unique perspective on cancer assessment. AI algorithms enhance PET-CT imaging by improving the visualization and interpretation of cancer's metabolic activity. This is particularly important for identifying metastatic or recurrent disease [23,32]. AI helps quantify tumor activity and assess response to treatment, facilitating personalized therapy adjustments [23,33]. Moreover, AI’s ability to integrate and analyze data from PET-CT, alongside other imaging modalities like CT and MRI, allows for multimodal data fusion that provides a comprehensive overview of the tumor's behavior and environment [23,26,33]. These features that AI provides when analyzing scans can lead to more informed clinical decisions [23,28,33]. When managing a patient with non-specific symptoms, an ENB may not be considered a primary differential diagnosis. Implementing AI in the radiological diagnostic process enables comprehensive analysis of tumor characteristics, patient health information, and other risk factors, thereby facilitating a confident diagnosis of an ENB. 

Histopathological Analysis

The integration of AI and ML into histopathological practices is transforming the precision and efficiency of cancer diagnostics. Histopathology, the examination of biological tissues to observe the appearance of diseased cells and tissues in very fine detail, is fundamental in diagnosing and subtyping cancers [26,33]. AI and ML technologies significantly refine the diagnostic process in histopathology by automating the analysis of tissue samples [23,26,28]. Traditional histopathological examination involves manually scrutinizing tissue slides under a microscope, a process that is highly skilled but subject to human error and variability. AI-driven tools, particularly those employing deep learning algorithms, can analyze digital images of histopathological slides with high accuracy, reproducibility, and speed [23,26,28]. Like with radiological imaging, these algorithms are trained on vast datasets of annotated images, enabling them to detect subtle patterns and anomalies that might be overlooked by human eyes [26,28]. AI algorithms can identify and quantify features such as cell size, shape, the arrangement of cells, and the density of cell nuclei, which are critical markers of malignancy [23,26]. ML models are adept at recognizing complex patterns associated with specific types of cancer [23,28]. 

For instance, approaches that use a convolutional neural network (CNN) to extract features have successfully differentiated between benign and malignant tumors, achieving a breast cancer prediction accuracy of 99.86%, which surpasses the ability of the human eye to detect or differentiate [26]. Sumaiya et al. showcased how CNNs can be utilized for feature detection in histopathological slide analysis, and how classification of the pathology can be achieved using a fully connected artificial neural network (ANN) [26]. An ANN is a method in AI that enables computers to process data in a way that mimics the structure and function of biological neural networks in the human brain. ANNs can develop interconnected nodes, known as neurons, which link incoming information to previously established data, or its “memory.” When the human brain encodes new information, it either stores it in memory or connects it to previously learned information to build a foundation of knowledge. ANNs strive to mimic this process by creating new nodes for novel information. This establishes a comprehensive neural network of information, enabling significant clinical applications. For ENBs, CNNs can be utilized to identify specific histopathological features of early-stage tumors, while ANNs can support classification and diagnosis by linking new histopathological data to previously stored ENB images and information in its neural network. 

Additionally, following these principles, an ML model can be developed to distinguish between an ENB and other sinonasal malignancies that present with similar symptoms. Other differential diagnoses to consider include nasal and paranasal squamous cell carcinoma, sinonasal polyposis, and choanal polyps. The challenge in diagnosing ENB promptly arises from its similar presentation to other sinonasal malignancies. However, an ML model may enable histopathological distinction of ENBs from other malignancies, thereby helping physicians accurately identify ENBs. An earlier diagnosis enables patients to receive timely and appropriate care. These CNN-based approaches excel in image pattern recognition, aiding the analysis of histopathological slides and enhancing the interpretation of various medical imaging modalities, including MRI, CT, and PET-CT scans [23,26,28].

Like all ML models, CNNs begin by preprocessing a dataset of images to reduce dimensions and eliminate redundancy, thereby enhancing efficiency without losing critical data, and ultimately normalizing the dataset [26]. CNNs inherently have the ability to detect and learn important features from the raw pixel data of images, refining these abilities through the backpropagation of errors during training [26,28]. Unlike traditional methods where features need to be manually extracted and defined, many ML models, such as CNNs, can learn these features directly from the data [26,28]. For example, features like the shape and texture differences between benign and malignant tissues are learned during training.

The network adjusts its filters to recognize patterns such as irregular nuclei or changes in the cytoplasm that are indicative of malignant cells [34]. The network comprises convolutional layers, which apply filters to detect important visual patterns such as edges or specific textures. These are followed by pooling layers that reduce the spatial size of the feature maps [26,34]. This reduction decreases the number of parameters and computations needed, thus simplifying the network while retaining the critical features necessary for classification. After several convolutional and pooling layers, the feature maps are flattened and fed into a fully connected ANN [26]. This part of the CNN acts as a classifier. It uses the learned features to determine whether the tissue is benign or malignant. The fully connected layers analyze the weighted feature map from the final pooling layer to compute the output, where the network makes its final decision, classifying the tissue as benign or malignant based on the learned features and patterns [26]. The system is trained with a substantial dataset, using the differences between the network's predictions and actual data to adjust and improve its predictive accuracy continuously [26]. This streamlined, automatic approach allows CNNs to effectively differentiate and classify cells and tissues based on learned visual patterns without manual feature extraction [35]. This highlights the efficiency of ML in medical imaging analysis and histopathological analysis, leveraging its ability to process complex image data, learn significant features autonomously, and perform accurate classifications, all essential for effective cancer detection.

Furthermore, accurate cancer subtyping is crucial for determining the appropriate treatment protocol, predicting disease progression, and improving patient outcomes [23,28]. AI and ML excel in classifying cancers into their respective subtypes based on histological patterns and genetic data integrated from other sources like genomic analysis [23,33]. AI models that analyze histopathological images can be integrated with genomic data analysis to enhance the identification of tumor subtypes with specific genetic profiles. This integrative approach helps in understanding the tumor's behavior and potential response to various treatments. By correlating certain visual features with clinical outcomes, AI can also help predict the aggressiveness of the tumor and the likelihood of recurrence, aiding in prognosis determination [23,33].

AI and ML in ENB management

The integration of AI and ML in cancer management has revolutionized the approach to treatment planning, prognostication, and surveillance. These technologies enable more precise, personalized, and proactive management of cancer, enhancing outcomes and the quality of care [23,28]. AI and ML significantly enhance treatment planning through decision support systems and predictive models that optimize surgical approaches, radiation therapy, and chemotherapy regimens [23]. AI-driven tools analyze vast amounts of imaging data, leveraging radiomic features to accurately characterize tumors, helping clinicians determine the most effective treatment strategies tailored to individual patient profiles [20,23,28]. For example, radiomic features from imaging scans have been shown to predict responses to specific treatments like gamma knife radiosurgery or radiation therapy, thereby guiding decisions on the most appropriate and effective interventions. Building on this, AI models further enhance treatment planning by providing detailed visualizations and precise segmentations of tumors, allowing for more accurate surgical interventions [23]. These models can delineate tumor boundaries more accurately than traditional methods, ensuring surgeons remove as much of the tumor as possible, while sparing healthy tissue. Additionally, ML algorithms, specifically CNNs, can be used to optimize radiation doses and sculpt radiation beams to conform closely to the tumor shape, minimizing exposure to adjacent normal tissues [23,28]. This precision reduces side effects and improves treatment efficacy. 

Prognostic models developed through AI integrate clinical, radiological, and molecular data to predict outcomes and guide therapeutic strategies [23,27,29]. By analyzing patterns across these data types, AI-based radiomics can potentially forecast disease progression, survival rates, and recurrence with greater accuracy than conventional methods [23,28,29]. AI and ML models use data from past patient outcomes combined with current patient data to predict survival times and progression-free intervals [29]. These predictions help patients and physicians make informed and enhanced decisions about treatment planning. Furthermore, AI enhances the understanding of tumor biology by analyzing genetic, transcriptomic, and epigenetic data, which plays a crucial role in predicting how tumors will respond to various treatments and in identifying potential therapeutic resistance mechanisms [23,33]. AI and ML models are central to the integration of genomics, pathomics, and radiomics data. By integrating imaging data with clinical and molecular insights, AI provides a comprehensive view of the patient's condition and a deeper understanding of the complexities of brain tumors at various scales and modalities [23,33]. Analyzing the genetic makeup of tumors through techniques like next-generation sequencing (NGS), AI helps identify specific genomic alterations and biomarkers [27]. This information is vital for molecular subtyping, which enables the creation of personalized treatment plans tailored to the unique genetic profile of each patient’s tumor [20]. This approach results in better-informed prognostic assessments and enhances the efficacy of treatments [23,33].

Automated surveillance algorithms are another critical application of AI in cancer management, particularly for the early detection of recurrence and the monitoring of treatment response [23,33]. These algorithms continuously analyze patient data, including imaging and molecular markers, to detect signs of disease progression or recurrence at the earliest possible stage and enable timely interventions. To achieve this, AI algorithms can detect subtle changes in imaging that may indicate recurrence before they become clinically apparent, facilitating early and potentially more effective treatment [25]. Additionally, AI systems can assess how tumors respond to treatment over time by monitoring changes in tumor size and metabolic activity. This provides real-time insights that can lead to timely adjustments in therapy, which traditionally relies on subjective and time-consuming methods. This approach not only accelerates the monitoring process but also improves its accuracy, ensuring that adjustments to treatment plans are made promptly and appropriately based on objective data [23,33]. Ultimately, the implementation of AI and ML models in the management of various cancers, including ENBs, can significantly improve patient outcomes.

Discussion

Challenges and Considerations

The deployment of AI and ML in healthcare introduces a range of challenges and considerations that need to be addressed to fully realize the benefits of these technologies. Among these challenges are issues related to data quality and standardization. One of the primary concerns in deploying AI and ML models in healthcare is the heterogeneity of data [28,33]. Medical data can come from multiple sources, including electronic health records, imaging, and genetic data, and often appear in various formats that vary in quality and granularity. This diversity can lead to inconsistencies that affect how AI models are trained and how they perform. For instance, image data from different MRI machines or settings may differ, affecting the consistency of the input data for AI models. Ensuring that these models can handle and interpret diverse data types is crucial for their accuracy and performance. Related to data heterogeneity, interoperability issues arise when different healthcare systems and technologies struggle to communicate and share data efficiently [28]. This lack of interoperability can impede the effective training and deployment of AI models, as they may not access comprehensive data across systems. Establishing common standards and protocols can alleviate these issues, enhancing the seamless integration of AI tools across various platforms.

AI and ML models can only be as good as the data they are trained on. If the training datasets are not diverse or if they contain biased data, the resulting models will likely perpetuate these biases, leading to poor performance in underrepresented groups [28,32,36]. Addressing this requires strategies such as data augmentation, oversampling, and undersampling to ensure a balanced representation in the training data [28]. Furthermore, ongoing efforts to analyze model performance across different population subgroups are essential to identify and mitigate potential biases [28,32,36]. Additionally, the sharing of validated data should be incentivized to enhance the accurate and beneficial development of AI and ML models in the medical field. ENBs present a unique challenge in this context due to their rarity. With limited data available, training ML models to achieve sufficient accuracy becomes problematic. ML modules require vast datasets to be accurate and unbiased, and the scarcity of ENB cases means that data might be insufficient for robust model training. This limitation underscores the need for innovative data-sharing initiatives and collaborative research efforts to construct larger, more extensive datasets that can support the effective training of AI models in rare cancers like ENBs. It should be encouraged that health professionals who treat and manage ENBs should compile a comprehensive pathologic report to better characterize the nature of the tumor, and this can be provided to machine learning models in order to build a foundational network of data to make the recognition of ENBs efficient. 

While the diversity and quality of data are foundational to the efficacy of AI and ML models in the diagnosis and management of ENBs, it is essential to acknowledge that even with optimal data, these models have inherent limitations. No current AI system can perfectly predict or diagnose without errors, as machine learning algorithms inherently approximate and simplify complex medical realities. They may also behave unpredictably or produce results that, while statistically valid, may not be clinically relevant or safe in specific ENB cases. This acknowledgment of the imperfection of AI, even under ideal conditions, is crucial for managing expectations and ensuring careful oversight in clinical applications. As such, continuous evaluation of AI-driven outcomes and integration of human oversight remain essential to address these intrinsic limitations and safeguard patient health.

Besides challenges related to data quality and standardization, the implementation of AI and ML models in healthcare may also encounter regulatory and ethical considerations to ensure that these technologies are used safely and effectively. The integration of AI in healthcare raises significant concerns about data privacy [24,28,33]. Ensuring the security of patient data against unauthorized access is paramount, especially when dealing with sensitive medical information. Regulations such as the General Data Protection Regulation (GDPR) in Europe and the Health Insurance Portability and Accountability Act (HIPAA) in the United States provide frameworks for data protection, but with the global nature of data sharing and AI development, maintaining privacy across borders becomes more complex [24,28]. The opaque nature of many AI models, often referred to as the black box problem, complicates the process of obtaining informed patient consent [24,28,33]. Patients must understand how their data will be used, the risks involved, and the potential benefits. This is particularly challenging when it is difficult to elucidate how AI models make decisions. Efforts to increase transparency, such as through Explainable AI (XAI), are vital in making these processes more transparent and understandable to patients and healthcare providers alike [28]. Ensuring the transparency of AI algorithms is crucial for building trust and for clinical adoption. It is necessary for clinicians and patients to be able to understand how decisions are made, particularly in cases where AI is used to support or make vital diagnostic decisions. This transparency is also linked to accountability; it must be clear who is responsible when AI and ML models make errors or when they are involved in decisions that lead to patient harm [28]. Thus, it is essential to establish and apply explicit rules and policies for the use of AI in healthcare environments, focusing on accountability in decision-making and clarifying the roles of medical staff and AI and ML models [28]. Equally important is ensuring that these AI and ML models are affordable so that their beneficial and revolutionary impact on patient care can be realized without increasing existing disparities in the healthcare system.

While AI and ML have the potential to significantly enhance efficiency and decision-making in the diagnosis and management of ENBs, it is essential to preserve the human element in patient care to ensure that clinical judgments are appropriately integrated with technological insights. Therefore, reliance on AI should not overshadow the clinical judgment of healthcare professionals. As AI and ML continue to be integrated into the field of medicine, addressing these data quality, standardization, regulatory, and ethical considerations is crucial for leveraging their potential responsibly. Ultimately, balancing the innovative capabilities of AI with protecting patient welfare and privacy will be key to the effective and just use of AI in healthcare.

Future Directions

As medicine advances to provide better care for patients, technology will become an integral part of that care. A physician's role is to understand patients at a deeper level and utilize this understanding to inform their diagnosis, treatment, and management. Similarly, AI aims to enhance the understanding of patients’ pathologies and facilitate the storage of clinical information. Currently, AI and ML models aim to integrate genomic, transcriptomic, and proteomic data from patients with specific pathologies into a unified database. This database can then be utilized to identify specific risk factors and trends associated with these pathologies, helping physicians better understand the current health state of their patients. 

In the context of ENBs, genomic data can identify mutations or alterations in genes associated with cancer risk, progression, and response to treatment. AI algorithms can then analyze this data to predict a patient's likelihood of developing ENBs, guide treatment selection, and identify potential therapeutic targets. Transcriptomic data offer information on gene expression levels, indicating which genes are actively being transcribed into RNA molecules. By analyzing transcriptomic profiles, AI algorithms can identify gene expression signatures that are associated with different subtypes of ENBs, disease progression, and treatment response. Integrating transcriptomic data into decision support systems can help tailor treatment strategies to match the unique molecular characteristics of each patient's tumor. Proteomic data provide insights into the proteins expressed within cells, tissues, or bodily fluids. Proteomic analysis of ENBs can identify proteins associated with tumor development, metastasis, and drug resistance. Building on this, AI-driven analysis of proteomic data goes further by enabling the identification of novel biomarkers, significantly enhancing the diagnosis of ENBs, monitoring disease progression, and improving the prediction of treatment outcomes.

Many hospitals across the United States have adopted AI into their healthcare ecosystems, and this growing trend is expected to continue. For proper clinical implementation, hospital staff should be offered hands-on training modules on how to utilize various AI models. Additionally, the use of AI models should be encouraged in the diagnostic process. Several strategies that healthcare systems can utilize to better implement AI include upholding data integration and interoperability. This entails investing in robust data infrastructures to integrate various sources of healthcare data, including imaging, genomics, and electronic health records. Permitting interoperability between different systems enables AI algorithms to analyze comprehensive patient data, leading to more accurate outcomes. Also, healthcare teams can integrate AI into radiology departments, where it can play a preliminary role in analyzing medical images. This will assist radiologists in detecting abnormalities, quantifying disease progression, and predicting treatment outcomes. Additionally, hospitals should be encouraged to adopt an open-minded approach to employing AI-enabled clinical decision support systems. Hospitals can utilize these systems to assist healthcare providers in diagnosis, treatment planning, and patient management. These systems have been shown to analyze patient data in real time, provide evidence-based recommendations, and alert clinicians to potential risks or deviations from best practices.

## Conclusions

An ENB is a rare sinonasal tumor that originates in the olfactory region of the upper nasal cavity. Diagnosis of ENBs is challenging due to non-specific early-onset symptoms and often insignificant findings during physical exams. Unique symptoms and physical findings begin to appear in the later stages of the tumor’s development; however, by this point, the tumor progresses to a stage that requires immediate treatment. With the emergence of AI and ML, healthcare systems are encouraged to adopt these innovative technologies to improve the diagnosis, treatment, and management of patients with rare tumor malignancies.
